# The summer of hydrogen sulfide: highlights from two international conferences

**DOI:** 10.1186/2045-9912-3-5

**Published:** 2013-02-25

**Authors:** John W Calvert

**Affiliations:** 1 Department of Surgery, Division of Cardiothoracic Surgery, Carlyle Fraser Heart Center, Emory University of Medicine, 30308, Atlanta, GA, USA

**Keywords:** Gasotransmitter, Hydrogen sulfide, Nitric oxide, Carbon monoxide

## Abstract

A great deal of interest has been paid recently to the hydrogen sulfide, the newest member of the gasotransmitter family. With the growing interest in the biology of H_2_S, the need for meetings and conferences dedicated solely to the field of H_2_S has also grown. In 2009, scientist from around the world met in Shanghai, China for the first time to discuss the physiological relevance of H_2_S. In 2012, two conferences were organized to bring scientists, clinicians, and industry representatives together to discuss the latest breakthroughs concerning the emergent field of H_2_S. The following is a summary report of The First European Conference on the Biology of Hydrogen Sulfide and the Second International Conference on Hydrogen Sulfide Biology and Medicine.

## Introduction

Gasotransmitters are small, labile, endogenously-produced gaseous molecules that play important roles in cellular signaling [1]. Hydrogen sulfide (H_2_S) is the newest member of the gasotransmitter family. Historically, it was considered to be highly toxic and hazardous to the environment [2]. However, H_2_S has recently emerged as a critical physiological gaseous signaling molecule that is produced enzymatically in all mammalian species at low micromolar levels via the action of cysteine metabolic enzymes: cystathionine-γ-lyase (CSE), cystathionine-β-synthase (CBS), and 3-mercaptopyruvate sulfutransferase (3-MST) [3]. Because of this discovery, the biological and physiological role of H_2_S has been re-evaluated. As such, an extensive amount of work has been conducted over the last several decades and has led to the discovery that H_2_S possesses a number of physiological actions.

In 1989, while investigation the effects of acute H_2_S poisoning in the brain, Warenycia *et al.*[4] first reported that H_2_S was produced endogenously. Soon after this discovery, other investigators started to take a closer look at H_2_S and evidence soon emerged to demonstrate its physiological importance. For instance, Skrajny *et al.*[5] found increased levels of serotonin and reduced levels of norepinephrine in the frontal cortex of a rat when chronically exposed to 20 ppm of H_2_S and in 1996, Abe *et al.*[6] suggested that H_2_S was an endogenous neuromodulator, as they showed that physiological concentrations of H_2_S enhanced *N*-methyl-D-aspartate (NMDA) receptor-mediated responses and aided in the induction of hippocampal long-term potentiation. Shortly after, Hosoki *et al.*[7] reported that an H_2_S-producing enzyme was present in the vasculature and proposed that H_2_S may be an endogenous smooth muscle relaxant. Since these initial offerings, studies have begun to characterize its biological profile and therapeutic potential. Over the last several years, a great amount of work by investigators all over the world have led to the suggestion that H_2_S is a powerful signaling molecule with a diverse physiological profile. Moreover, because of its physiological actions, H_2_S has been reported to protect the heart, brain, liver, kidney, and lungs against a variety of pathological stimuli, including ischemia/reperfusion (I/R) injury [8-17].

With the growing interest in the biology of H_2_S, the need for meetings and conferences dedicated solely to the field of H_2_S has also grown. In 2009, scientists from around the world met in Shanghai, China for the first time to discuss the physiological relevance of H_2_S. In 2012, two conferences were organized to bring scientists, clinicians, and industry representatives together to discuss the latest breakthroughs concerning the emergent field of H_2_S. The following is a summary of the highlights from those two meetings.

### First European conference on the biology of hydrogen sulfide

The First European Conference on the Biology of Hydrogen Sulfide was held at Smolenice Castle on the outskirts of Bratislava, Slovakia from June 15 to 18, 2012. The Institute of Molecular Physiology and Genetics from the Slovak Academy of Sciences sponsored this conference. Dr Karol Ondrias from the Slovak Academy of Sciences served as the Conference Chairman and Dr Matthew Whiteman from the University of Exeter served as Honorary Chairman and Chairman of the International Advisory Committee. Together with the rest of the members of the International Advisory Committee, Drs Ondrias and Whiteman organized an outstanding scientific program consisting of 12 plenary lectures, 34 short oral presentations, and 15 poster presentations.

The meeting started with a Keynote Lecture delivered by Dr Rui Wang from Lakehead University in Thunder Bay, Ontario, Canada. In his lecture, Dr Wang discussed several recent findings regarding where inside the eukaryotic cell H_2_S is produced and how this intracellular compartmentalization is realized. He also discussed the functional consequences of H_2_S production in response to difference stimuli. Dr Wang’s lecture was followed by a very nice welcome reception where the attendees were inundated with local Slovakian cuisine.

Day 2 of the conference began with a session focused on the effects of H_2_S on heart and vascular physiology. Dr Y.Z. Zhu from Fudan University in Shanghai China began the session by detailing recent work on the involvement of H_2_S in the cardiovascular disorder processes and the prospects of new therapeutic strategies based on the regulation of endogenous H_2_S production [18]. This session was highlighted by Dr David Lefer from Emory University in Atlanta GA who presented recent work investigating the role of cystathionine gamma lyase derived H_2_S in the development of pressure-induced heart failure. He also presented novel and exciting data regarding the effects of oral H_2_S therapy on preventing cardiac dysfunction following the induction of heart failure.

There is emerging evidence that members of the gasotransmitter family can interact with each other. For instance, NO and H_2_S have been suggested to collaborate in regulating vascular homeostasis and vasodilation. Additional evidence suggests that NO can increase CGL activity acutely, and that chronic exposure to NO up-regulates CGL expression. Moreover, at low concentrations H_2_S has been shown to enhance the release of NO from vascular endothelium and increase the vasorelaxant effect of the NO donor sodium nitroprusside [19]. Furthermore, a 10 fold increase in H_2_S levels have been reported with administration of NO donors [20], suggesting a synergistic relationship between NO and H_2_S. Dr Andreas Papapetropoulos from the University of Patras in Greece detailed very exciting and new results that further demonstrate the interdependence of the gasotransmitters on each other. Specifically, he provided the audience with compelling evidence that angiogenesis and vasodilatation require the simultaneous production of H_2_S and NO, suggesting that both are mutually required for the control of vascular function [21].

Day 2 concluded with two afternoon sessions. The first session focused on the ability of H_2_S to regulate respiratory and gastrointestinal function. Dr John Wallace from McMaster University in Hamilton, Ontario, Canada discussed his group’s latest findings regarding the ability of novel H_2_S releasing anti-inflammatory drugs to alleviate the development of gastrointestinal (GI) ulcers [22]. The last session of Day 2 focused on H_2_S donors and was highlighted by Dr Mathew Whiteman’s presentation on the use of H_2_S donors to treat inflammatory joint disease.

Dr Csabo Szabo from the University of Texas Medical Branch in Galvaston, TX opened the morning session on Day 3 by discussing the role of H_2_S deficiency in the pathogenesis of diabetic vascular complications. In his presentation he provided the audience with compelling data suggesting that H_2_S deficiency is emerging as a significant pathogenic mechanism in diabetic complication, while H_2_S supplementation has the potential to be a novel therapeutic approach [23]. The session on H_2_S and diabetes was followed by a session regarding the interaction of H_2_S with the mitochondria. This session was highlighted by Dr Katalin Modis’ (University of Texas Medical Branch in Galvaston, TX) presentation detailed her exciting work demonstrating that H_2_S has a physiological role as a positive modulator of mitochondrial electron transport that is dependent on a basal activity of the Krebs cycle [24].

In the afternoon, Dr Hideo Kimura from the National Institute of Neuroscience in Tokyo, Japan led off a very interesting and stimulating session on the synthesis of hydrogen sulfide. In his talk, Dr Kimura detailed his group’s efforts to identify the endogenous reducing substances (thioredoxin and dihydrolipoic acid) that facilitate the production of H_2_S from 3-mercaptopyruvate sulfurtransferase [25]. This was followed by a very stimulating presentation from the Ondrias group detailing their work suggesting that H_2_S induces the release of NO from nitroso groups. Day 3 ended with a very informative session regarding the interaction of H_2_S with different ion channels.

The Final Day of the conference ended with several talks discussing the latest findings regarding the role of H_2_S in the setting of cellular stress and cell proliferation. Additionally, awards were handed out at the conclusion of the conference to several young investigators.

### The second international conference on H_2_S biology and medicine

With the momentum gained at Smolenic Castle in Bratislava, scientists, clinicians, and industry representatives from around the world descended on Atlanta, GA from September 20 – 22, 2012 to attend the Second International Conference on H_2_S Biology and Medicine. Dr David Lefer from Emory University in Atlanta, GA organized this conference. Together with the rest of the member of the Organizing Committee, Dr Lefer planned a well-balanced scientific program detailing the latest findings concerning the chemistry and biology of H_2_S. The final program consisted of 30 plenary lectures, 4 short oral presentations, and over 75 poster presentations.

Dr Solomon Snyder from Johns Hopkins University in Baltimore, MD delivered the keynote lecture to start the conference. In his lecture, Dr Solomon eloquently discussed his group’s efforts to characterize sulfhydration, which is the mechanism by which H_2_S modifies reactive cysteine residues on targeted proteins. Although analogous to protein nitrosylation, sulfhydration is substantially more prevalent and usually increases the catalytic activity of targeted proteins [26].

Dr Snyder’s lecture was followed by several presentations on the biology and chemistry of H_2_S. Highlights from this session included Dr Nicholas Tonk’s (Cold Spring Harbor Laboratory) presentation on his recent work detailing of the effects of H_2_S on the endoplasmic reticulum stress response. Specifically, he presented data demonstrating that H_2_S reversibly inhibits the activity of protein tyrosine phosphatase 1B, which promotes the activity of protein kinase-like endoplasmic reticulum (ER) kinase (PERK) during the response to ER stress [27]. With the growing interest in the physiological and biological role of H_2_S, there is a need for better detection methods for H_2_S levels and signaling. This was a constant point of discussion throughout the meeting. As such, Dr Ming Xian’s (Washington State University in Pullman, WA) presentation on new chemical tools for H_2_S research was of particular interest. In his talk, Dr Xian provided evidence that newly developed fluorescent probes provide good selectivity and sensitivity for H_2_S [28].

The afternoon session of Day 1 was dedicated to discussion on the role that H_2_S plays in the development and treatment of cardiovascular disease. Dr Frank Sellke from Brown University in Providence, RI led off this session by discussing his labs previous work investigating the impact of H_2_S on myocardium in the setting of cold crystalloid cardioplegia and cardiopulmonary bypass [29]. In his talk, Dr Sellke provided compelling evidence that treatment with H_2_S offered myocardial protection via attenuation of caspase-independent apoptosis and autophagy in a porcine model of cardiopulmonary bypass. Later in this session, Dr Suresh Tyagi from the University of Louisville in Louisville, KY summarized his groups’ findings regarding the effects of H_2_S on cardiovascular remodeling in response to heart failure [30]. Day 1 concluded with a reception and presentation of posters.

Day 2 of the conference featured three sessions. The first session of the morning was focused on H_2_S and the vasculature. Dr Rui Wang from Lakehead University in Thunder Bay, Ontario, Canada started the session with a nice introduction to the effects of H_2_S on the vascular endothelium. This was followed by Dr Guiseppe Cirino’s (University of Naples, Naples, Italy) presentation on H_2_S as an endogenous inhibitor of phosphodiesterases and how this affects vascular tone.

The second session of Day 2 was focused on the H_2_S and gastrointestinal diseases. Presentations in this session from Drs. John Wallace (McMaster University in Hamilton, Ontario, Canada), Mauro Perretti (Queen Mary University), Nathalie Vergnolle (INSERM), and Andre Buret (University of Calgary) detailed the latest findings regarding the role of H_2_S in the resolution and repair of the GI tract and detailed the latest developments in use of novel H_2_S releasing drugs to treat GI disorders.

The final session of Day 2 was concentrated on the role of H_2_S in shock and sepsis. This session began with a very informative presentation by Dr Konstantin Shatalin (New York University) in which he discussed the role H_2_S plays in mediating bacteria defense against antibiotics [31]. Specifically, Dr Shatalin provided evidence that inactivation of putative cystathionine β-synthase, cystathionine γ-lyase, or 3-mercaptopyruvate sulfurtransferase in *Bacillus anthracis, Pseudomonas aeruginosa, Staphylococcus aureus*, and *Escherichia coli* suppresses H_2_S production, rendering these pathogens highly sensitive to a multitude of antibiotics. Dr Fumito Ichinose from Harvard University in Boston, MA then detailed recent findings from his lab in which they demonstrated that inhaled H_2_S prevents inflammation and improves survival in the setting of endotoxemia by altering sulfide metabolism in mice [32].

The final day of the conference began with presentations selected from submitted abstracts. This forum gave deserving young investigators and trainees the opportunity to present their latest findings in a more formal setting. This session was followed by a second session on H_2_S and the vasculature. Dr Christopher Peers from the University of Leeds in Leeds, England highlighted this session with his presentation on the regulation of ion channels by H_2_S. Dr Chris Kevil from LSU-Health Sciences Center in Shreveport, LA delivered the last talk of the conference detailing his groups latest work on the actions of H_2_S in the setting of high limb ischemia.

## Summary and comments

In summary, the First European Conference on the Biology of H_2_S and the Second International Conference on H_2_S Biology and Medicine were both a tremendous successes. Each conference provided an excellent platform for the presentation and discussion of the latest findings regarding the biology and chemistry of H_2_S. In addition, both of these conferences served as a reminder that that is still so much about H_2_S that we do not know. In this regard, a number of important issues/questions were raised at each conference that will certainly drive research in the years to come. These include (1) the need to develop more sensitive ways to measure H_2_S, (2) the need to develop new drugs or delivery mechanisms to deliver sustained levels of H_2_S, and (3) the need to understand the molecular targets of H_2_S and the manner in which H_2_S modifies these cellular targets.

It was recently announced that there will be a Second European Conference in Exeter England from September 8–11, 2013. Dr Matthew Whiteman will serve as the Organizer. In addition, Dr Hideo Kimura will serve as the organizer for the next International H_2_S meeting. He announced at the conclusion of the Atlanta meeting that the next International conference on H_2_S will be held in Japan in 2014. For more information about this conference and other conferences related to H_2_S please log on to the European Network on Gasotransmitters website for the latest news and developments (http://www.gasotransmitters.eu/).

## Competing interest

All authors declare that they have no competing interest.

## Disclosures

John Calvert served as Vice Chairman of the Organizing committee for the Second International Conference on H_2_S Biology and Medicine.

## Sources of funding

Supported by grants from the American Diabetes Association (7-09-BS-26) and the National Institutes of Health National Heart Lung and Blood Institute (NHLBI) (5R01HL098481-03) to J.W.C. This work was also supported by funding from the Carlyle Fraser Heart Center (CFHC) of Emory University Hospital Midtown.
